# A pre-market interventional, single-arm clinical investigation of a new topical lotion based on hyaluronic acid and peptides, EGYFIL^TM^, for the treatment of pain and stiffness in soft tissues

**DOI:** 10.1186/s12891-023-06903-y

**Published:** 2023-10-02

**Authors:** Stefano Picotti, Luca Forte, Jo Serrentino

**Affiliations:** 1 Poliambulatorio Centro Sistema Salute, Varese, Italy; 2 Contrad Swiss SA, Via Ferruccio Pelli 2, Lugano, 6900 Switzerland; 3 International Institute of Clinical Ecology (IICE), Quebec, Canada

**Keywords:** Myofascial tension, Anti-inflammatory, Pain, Peptides, Hyaluronic acid, Musculoskeletal

## Abstract

**Background:**

Muscle pain and stiffness are strictly interconnected. Injuries frequently occur during sport activities, causing muscle pain, with or without stiffness, and require effective as well as fast-acting treatments. Topical products can be ideal for the treatment of such physical alterations as they are convenient and simple to use. In this study, it was investigated the application of a novel topical formulation, EGYFIL™, for the treatment of pain and stiffness due to muscle contracture, trauma, and/or overtraining. The lotion is composed of hyaluronic acid, a well-known ingredient for the pain alleviation, mixed with skin conditioning SH-Polypeptide-6 and SH-Oligopeptide-1, embedded in it.

**Methods:**

Twenty-six patients with pain and/or stiffness were enrolled. After a screening visit (Time 0, t0), patients were treated for the first time with the IP. The treatment consisted of topical application of the pain lotion. Level of pain and stiffness were measured with Numerical Rating Scale (NRS). Patients’ pain and/or stiffness were evaluated at t0 (prior to using the product), after three hours (t1), and after three days (t2) of treatment. Participants were free to apply and re-apply the product ad libitum over the course of the study period (3 days). Potential adverse events (AE) and tolerance were evaluated during each visit.

**Results:**

There was a 22% decrease in pain in the first three hours (*p* < 0.001), followed by an additional 20% decrease after three days (*p*=0.0873). Overall, there was a 42% decrease in pain over the three days of the study (*p* =0.001). Furthermore, a 24% reduction in stiffness in the first three hours (*p*=0.025) and a 38% decrease in stiffness over three days (*p* < 0.001) were observed. Reduction in pain and stiffness were neither age, nor sex dependent. No adverse effects were reported during the study.

**Conclusion:**

EGYFIL™ is safe and seems to reduce pain and stiffness in patients during the 3 days of treatment, already after 3 h from the first application.

**Trial registration:**

ClinicalTrials.gov ID: NCT05711953. This trial was registered on 03/02/2023.

**Supplementary Information:**

The online version contains supplementary material available at 10.1186/s12891-023-06903-y.

## Background

Stiffness is a prelude to musculoskeletal pain [1, 2]. Myofascial tone is characterized by ‘stiffness’ that progressively leads to unfavourable loading conditions that cause micro injury and other pathologies such as tendinopathy, osteoarthritis, enthesopathies as well as vascular tension and claudication that can cause pain [3–5]. The mechanobiological pathways relevant to myofascial tone encompass cell signals, as well as sensing of external forces (activity, sports actions), causing muscular contractions [6, 7]. In this sense, stiffness is a feature of pain or injury resulting from shear stresses and pressures.

Mechano-molecular pathways associated with myofascial tension can lead to musculoskeletal pain [8, 9]. Myofascial tension (from athletics, loading, etc.) occurs over the entire muscle fibre surface and generates actomyosin filaments, contributing to force (action) of the skeletal muscles [10–13]. Although stiffness is often a prelude to pain, it is most commonly a consequence of chronic myofascial tension. The pain from sports injuries or osteoarthritis usually starts with changes in myofascial tone, leading to tension and stress that then cause injuries and pain [5, 14–18].

Hyaluronic acid (HA) is a hygroscopic glycosaminoglycan often utilised for the treatment of articular pathologies due to the elastoviscous property conferred by the polymer: it is thought to increase the viscoelastic and shock absorbing properties of the synovial fluid while also reducing the inflammation and pain in joint diseases of the knee or hip, more typically in over-weight patients [19–24]. HA has been studied as both a therapeutic component, in its own right; as well as a carrier for topical application of other substances [25]. Considering its well-known safety and biocompatibility, it is largely used in medical practice. In order to promote its functionality and soothing properties, it was combined in the EGYFIL™ formula with skin-conditioning peptides. They contribute to the conditioning of skin tissue, allowing a better penetration of active ingredients into deeper dermal tissue when topically applied [26–28]. Peptides have been utilized in several cases such as bone healing [29–31], osteoporosis [32], cartilage regeneration [33, 34] and wound healing [35]. Peptides are derived from starting proteins that can encompass a number of regulating proteins such as growth factors [36], that contribute to a range of therapeutic effects [37–39].

In EGYFIL™, two specific peptides are included in the lotion to promote the functional relief of pain and stiffness. SH-Polypeptide-6, derived from the interleukin-10 (IL-10) starting protein: SH-Polypeptide-6 carries IL-10's anti-inflammatory activity by engaging signals that modulate NF-kB pathways, downregulating pro-inflammatory cytokines TNF, IL-6, IL-1 and IL-8 [40, 41]. Like its parent protein IL-10, SH-Polypeptide-6 inhibits protein tyrosine phosphatase 1B expression that can cause dysregulation of the energy metabolism of skeletal muscles causing pain and muscular spasms [42]. Unlike its parent protein that requires breakdown to achieve this function, SH-Polypeptide-6 floods the in situ area immediately upon application; this achieves a better circulating bioavailability within tissue right at the site of pain, thereby quickly regulating the pain mechanisms.

The other peptide in EGYFIL™, SH-Oligopeptide-1 is synthesized from starting protein Epidermal Growth Factor (EGF). In the connective tissue matrix, EGF inhibits kappa B (NF-κB) pathway I and protects osteoblasts from inflammation and oxidative injury [43]. SH-Oligopeptide-1 is a functional matricellular peptide [44] that highly contributes functional healing within dermal cells and the connective tissue matrix structures such as tendons, ligaments, and muscles [45]. It mainly functions to improve circulation, stimulating vascular and lymphatic channels and cell mobility during the repair mechanism following injury, thereby reducing swelling and its associated pain [46]. Stimulated by the inflammatory response, EGF-derived SH-Oligopeptide-1 accelerates dermal repair and vascularization and promotes the synthesis of growth factors [47]. Considering its activity on vascular pathways, it supports recovery in case of oedema and swelling that can occur in concomitance with an injury, improving circulation and promoting faster recovery from the mechanical trauma to myofascial structures [48].

This prospective pre-market, interventional, single arm investigation aimed to enrol participants with stiffness, inflammatory pain, or both stiffness and pain, to evaluate the effectiveness of the Investigational Product (IP), EGYFIL™, a topical lotion.

## Methods

### Patients’ enrolment and follow-up

The study was approved by the independent Ethics Committee of International Institute of Clinical Ecology (IICE), (Approval Number: i072021E). The study was conducted according to the ISO 14155:2020, Good Clinical Practice guidelines, laws regarding the use of personal data (EU 2016/679), local Italian laws (196/2003) and the World Medical Association Declaration of Helsinki. This clinical trial was registered on 03/02/2023 with the following ClinicalTrials.gov ID: NCT05711953.

Initial examination and assessment were performed by the PI immediately following consultation.

Inclusion and exclusion criteria are indicated in Table 1. A total of 26 healthy adult participants were enrolled for the study, male or female, > 18 years old.

**Table 1:** Inclusion and exclusion criteria

Inclusion criteria:	1. signed participants informed consent form (ICF); 2. male or Female, aged > 18 years at the time of the signature of ICF; 3. 3 to 10 rating according to the Numerical Rating Scale (NRS), applied in participants with muscle stiffness and/or pain due to muscle contracture, trauma, and/or overtraining;
Exclusion criteria:	1. use of analgesics within the 24 h prior to baseline visit (t0); 2. damaged skin in the area of treatment; 3. infective or prior inflammatory processes near the area of treatment; 4. ongoing cutaneous allergies; 5. serious and chronical pathological skin conditions (i.e., rosacea, psoriasis, vitiligo) including diagnosticated cancer with/without ongoing antitumor therapy; 6. allergy to lotion components (aqua (water), glycerin, caprylic/capric triglyceride, *Aloe barbadensis* leaf juice powder, *Simmondsia chinensis* (Jojoba) seed oil, phenoxyethanol, ammonium acryloyldimethyltaurate/vp copolymer, carbomer, tocopheryl acetate, tocopherol, benzoic acid, sodium hydroxide, sodium hyaluronate, dehydroacetic acid, ethylhexylglycerin, *Butyrospermum parkii* (shea) butter, sorbitol, SH-Polypeptide 6; SH-Oligopeptide-1 7. immune system illnesses; 8. uncontrolled systemic diseases; 9. known drug and/or alcohol abuse; 10. mental incapacity that precludes adequate understanding or cooperation; 11. participation in another investigational study.

The maximum time of treatment for each enrolled patient was 3 days. Following an explanation of the aims of the study, patients that met all the inclusion criteria and none of the exclusion criteria, after having signed the informed consent form (ICF), entered the screening phase during which the baseline tests was conducted. At the baseline visit (t0), according to the instructions for use (IFU) and the judgement of the principal investigator (PI), the enrolled subjects were treated for the first time with the IP. The treatment consisted of topical application of the pain lotion immediately following the baseline visit. Primary endpoints, pain and stiffness, were both assessed through the use of a numerical rating scale (NRS) where zero corresponded to no pain/stiffness, and 10 represented the maximum possible pain/stiffness. Participants were asked about their pain and/or stiffness prior to using the product (t0), after three hours from the first treatment (t1), and after three days (t2) through phone contact and by means of NRS for pain and a questionnaire (Supplementary File 1). Participants were free to apply and re-apply the IP as desired. Time until relief was felt, was recorded as was the frequency of application. Potential adverse events (AE) and tolerance were evaluated during each visit.

### Treatment with EGYFIL™

EGYFIL™ is a novel topical product formulated as a water and glycerine-based lotion, containing sodium hyaluronate, a peptide mixture and some botanicals inside a 50 mL tube. The composition of the EGYFIL™ is reported in Table 2.

**Table 2:**
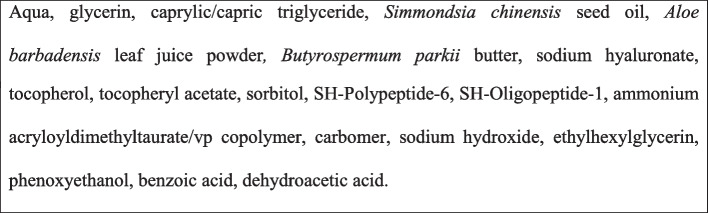
Composition of EGYFIL™

### Statistical analysis

Analyses were performed comparing each of the three timepoints with each other. Distribution of data was tested for normality by D’Agostino-Pearson tests. According to the result of this test, one-way ANOVA test with Tukey’s post hoc test were used to analyse the differences in the NRS between study visits. Unpaired t-tests were used to compare non-matched groups (different age brackets, sexes). *P* values < 0.05 were considered statistically significant. Data were analysed using Prism software v9.4 (Graphpad Prism, La Jolla, CA).

## Results

**Fig. 1:**
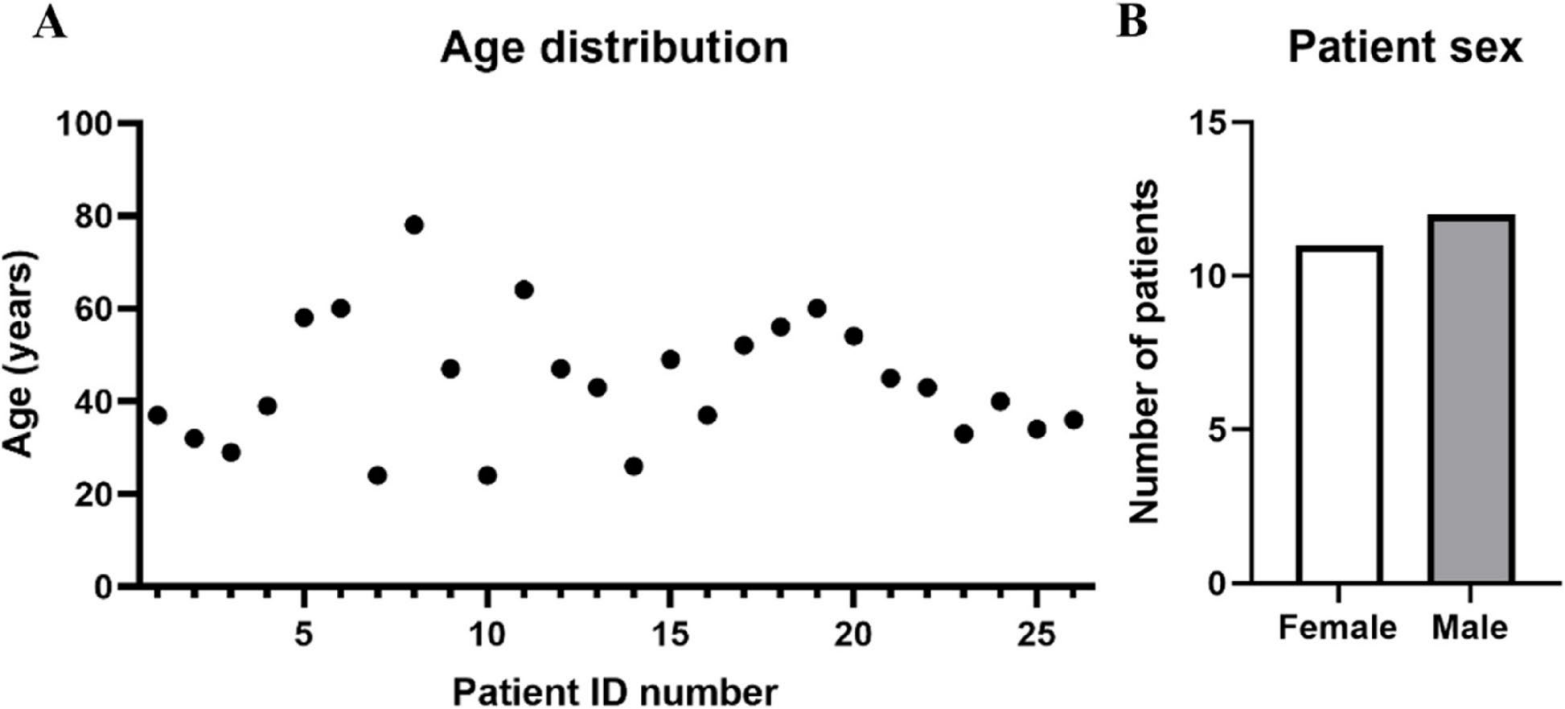
Characteristics of the enrolled population. **A** Patients ID number (abscissa) and relative age (ordinate). **B** Patients sex distribution

In the study, 26 patients were enrolled. All 26 enrolled patients completed the study. However, 3 patients used other analgesic products during the course of the study and were therefore excluded from the protocol set. Of the 23 remaining patients, 12 (52%) were male and 11 (48%) were female (Fig. 1A), and the mean age was 44 years old (ranging from 24 to 78 years old, Fig. 1B). There were no adverse effects noted during the study.

**Fig. 2:**
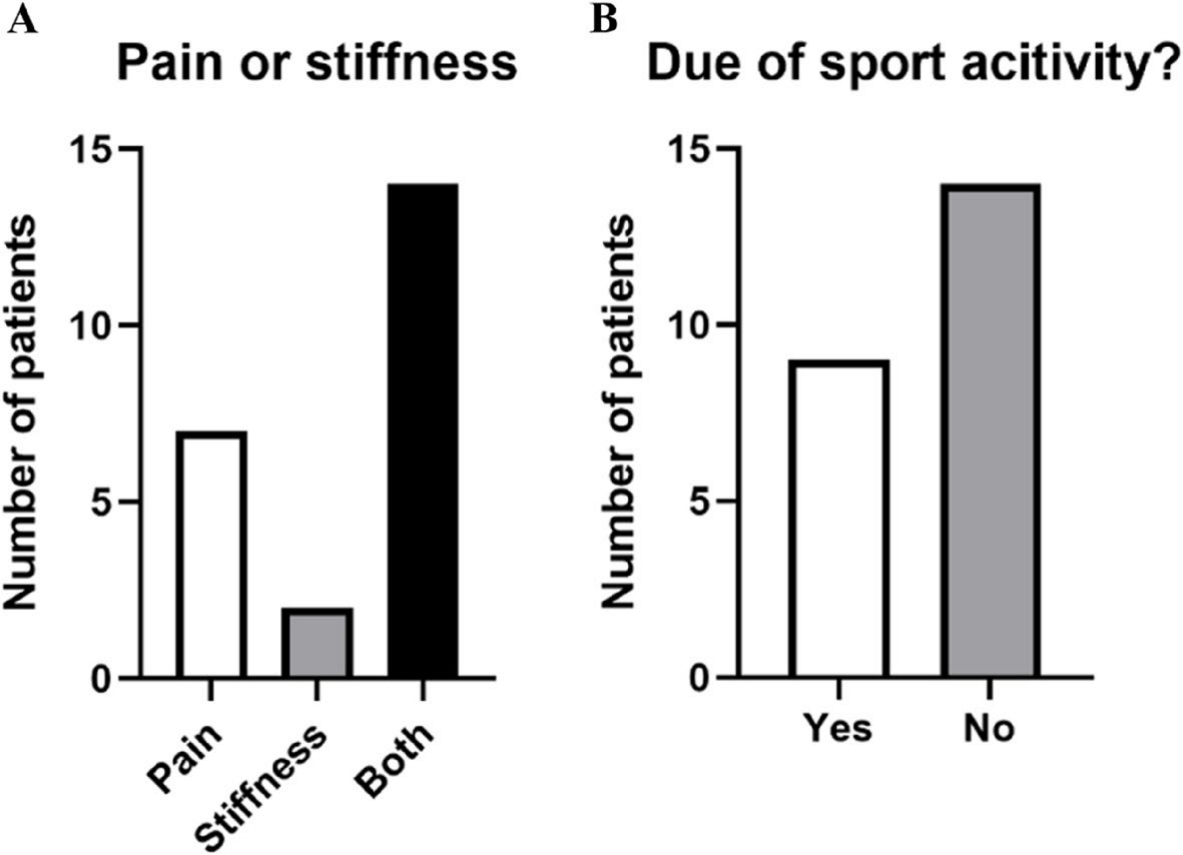
Description of patient’s symptoms and their causes at the t0. **A** Distribution of the number of patients affected by pain, stiffness, or both, at the time of the visit 1. **B** Representation of the number of patients suffering from complaints due to sports or not

Most of the 23 patients, 14 (61%), complained of both pain and stiffness, with 7 (30%) complaining of pain only, and just 2 (9%) participants with only stiffness (Fig. 2A). 9 patients out of 23 (35%) stated that their complaint was due to sports activities (Fig. 2B).

**Fig. 3:**
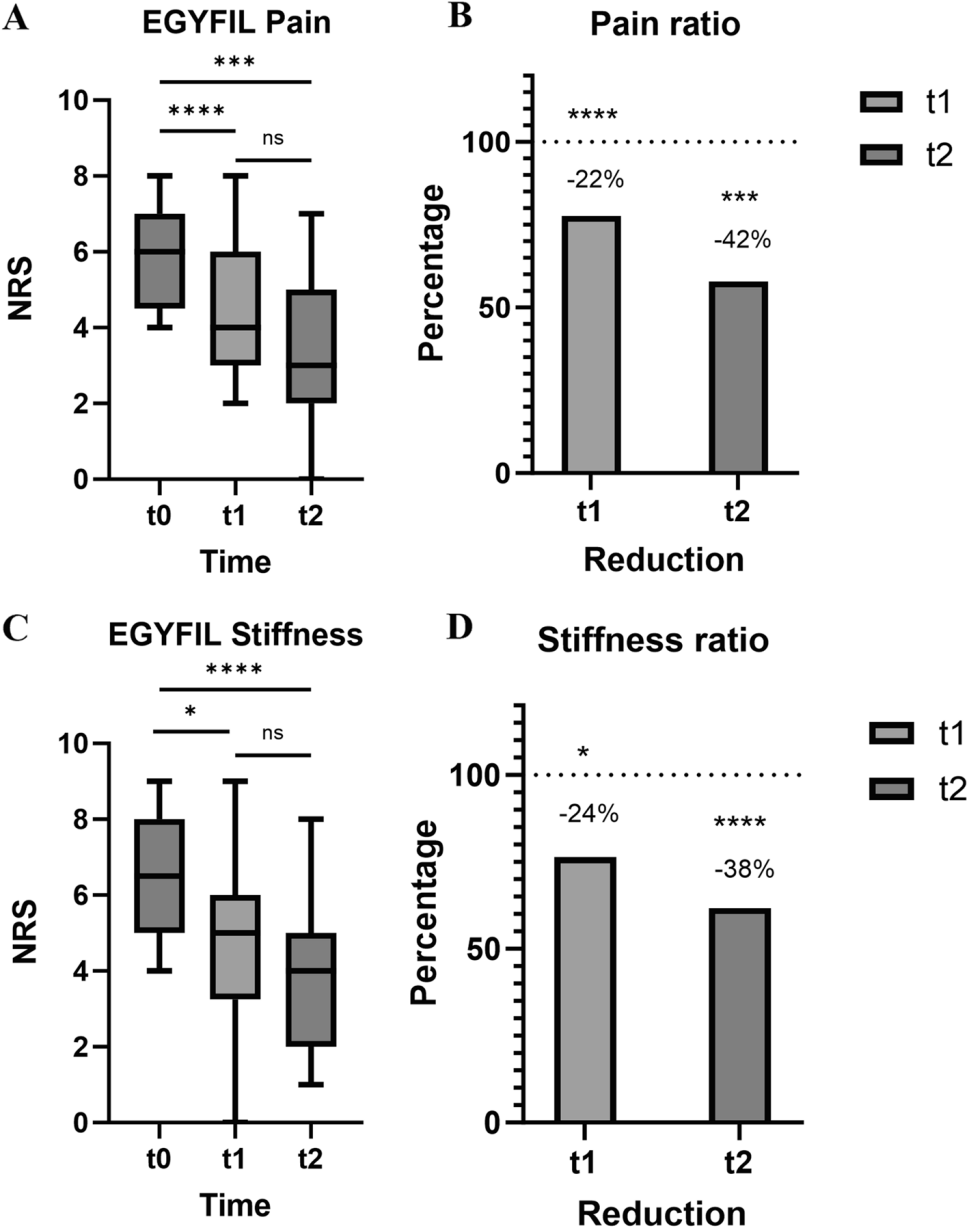
**A**, **B**. Mean NRS pain at t0, after 3 h (t1) and 3 days (t2) (**A**) and ratio Vs t0 (**B**). **C**, **D** Mean NRS stiffness at t0 and after 3 h and 3 days (**C**) and ratio Vs t0 (**D**)

NRS pain and stiffness scores are listed on Supplementary Table 1. The initial mean pain score was 5.8 which decreased to 4.5 after three hours and to 3.3 after three days (Fig. 3A). These scores indicate a 22% decrease in pain in the first three hours (*p* < 0.001), followed by an additional 20% decrease after three days relative to 3 h (*p* = 0.0873). Overall, there was a significant (*p* = 0.001) 42% decrease in pain over the three days of the study (Fig. 3B). The other endpoint, stiffness, was also reduced over the course of the study: from an initial mean score of 6.4 at T0 to 4.9 after three hours, and then to 3.9 after 3 days (Fig. 3C). These reduced scores in stiffness were statistically significant (*p* = 0.025, *p* < 0.001) and correspond to a 24% reduction in stiffness in the first three hours and a 38% decrease in stiffness over three days (Fig. 3D).

**Fig. 4:**
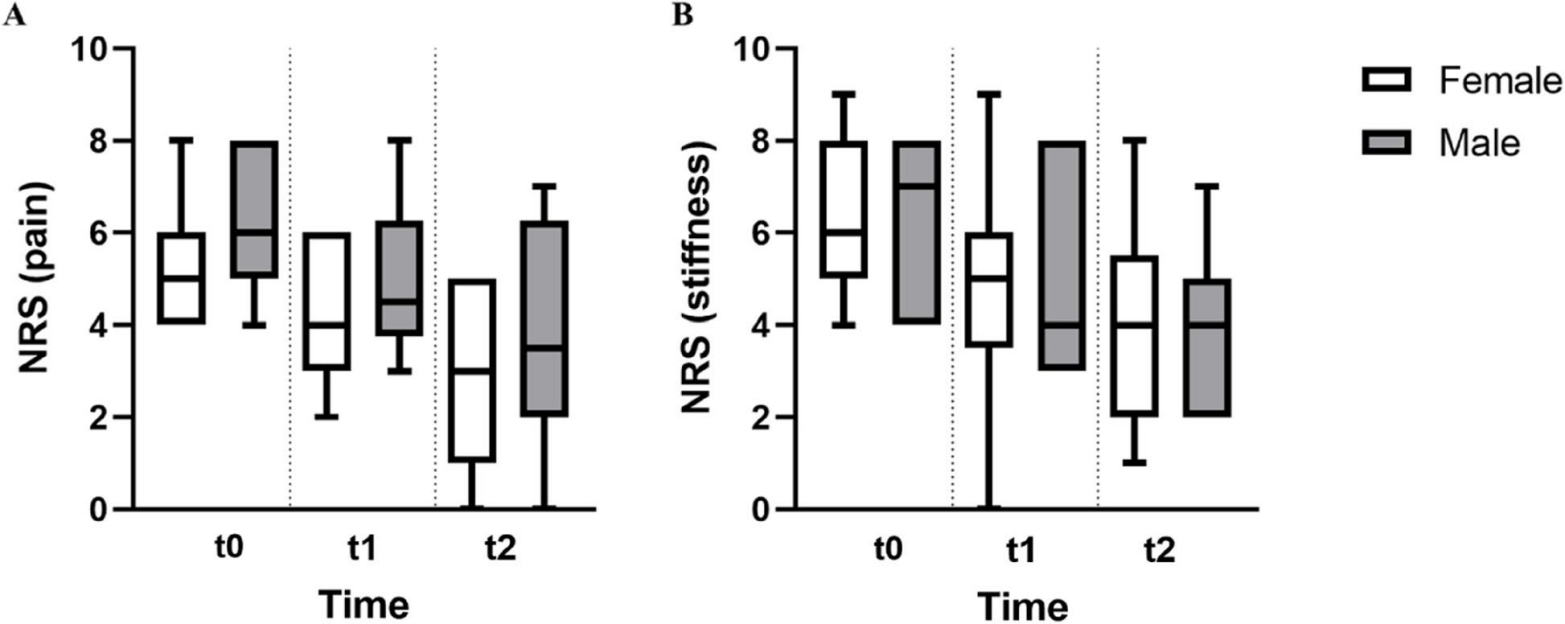
Distribution of pain (**A**) and stiffness (**B**) related to the sex of patients

The differences in the two endpoints, pain and stiffness, as assessed by NRS were neither sex nor age-dependent (Fig. 4. Age data not shown).

**Fig. 5:**
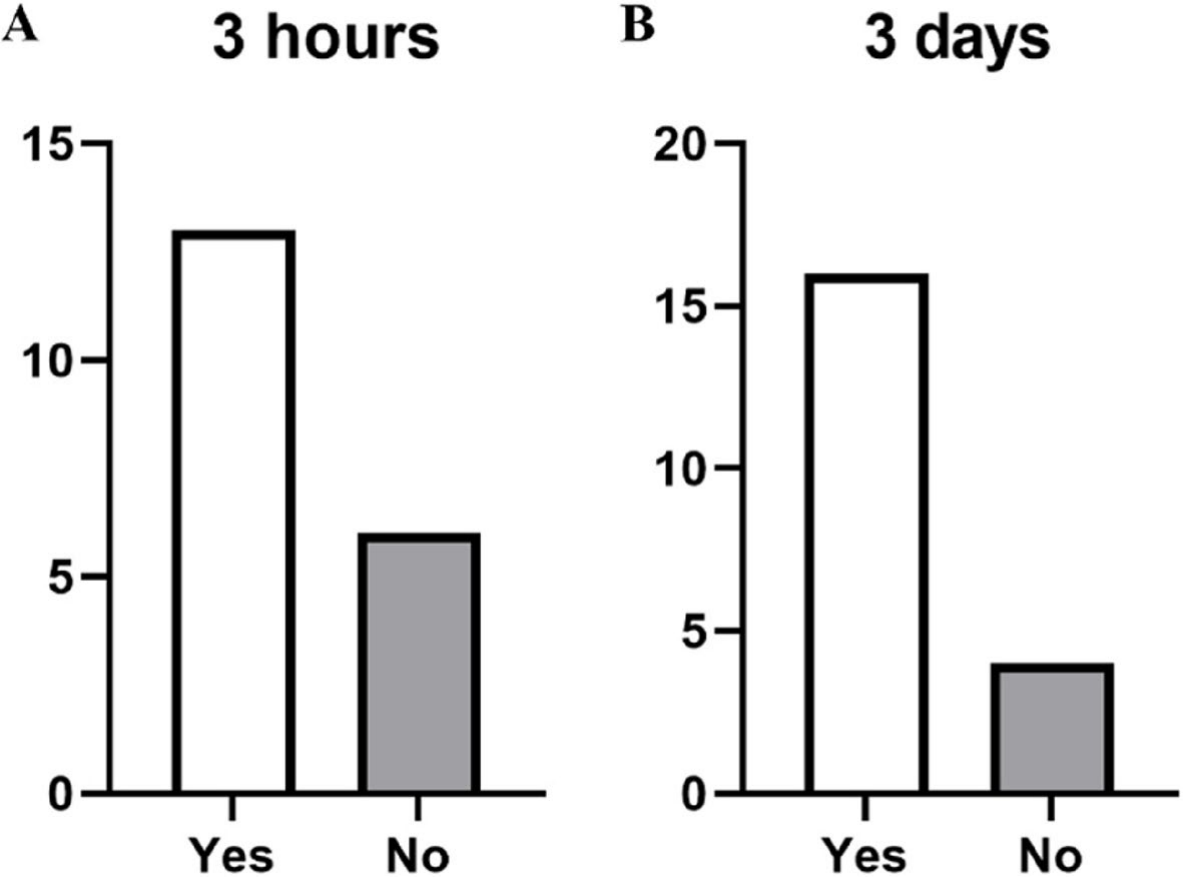
Sustained relief after 3 h (**A**) and 3 days (**B**) reapplication of the product

When asked at both 3-h and 3-day intervals, most patients reported that reapplying the product maintained the initial symptom relief (Fig. 5). There was no significant difference between the initial pain or stiffness levels of patients which indicated that repeated application of the product provided sustained relief from either stiffness or pain. However, patients that indicated that reapplication provided sustained relief from pain had a significantly lower NRS score for pain at the 3-day time point than patients that indicated no relief (2.6 Vs 5.8, *p* < 0.001).

There was a weak, but positive correlation between mean response (mean of 0-3 h and 3 h-3d responses) and satisfaction score (R^2^ = 0.13).

## Discussion

Overall, the product was well tolerated and favourably received by the participants of the study; there were no adverse reactions.

The 3-hours timepoint was selected for the speed of evaluation in a clinical setting. Furthermore, the study design was a real-time evaluation in the established setting of a sport practice, and 3 h is the timeline established within the parameters of the on-site event. The 3-days timepoint was used to evaluate the need for further administration of the product, if any, which also yielded initial data to further explore the efficacy and use of the product.

Pain was significantly decreased over the course of the study revealing a mean decrease in pain of 42% of initial levels after 3 days. In the short-term, pain was also decreased significantly by 22%. These decreases were not dependent on the sex or age bracket of patients (when patients were grouped by decade of birth). A robust response was seen in all groups. Similar results were seen for the reduction of stiffness at both 3 h (24%) and 3 days (38%) timepoints. Although it is not a standard scale to evaluate the stiffness, the use of NRS to assess this condition is not a completely novelty: it was already used to measure stiffness following small molecule therapy and tenosynovial giant tumor [49, 50] and rheumatic diseases [51, 52]. Stiffness is also quantified numerically as a sub-parameter of standard questionnaires to evaluate pain and joint mobility, like the WOMAC index [53].

Concerning the pain, the effectiveness of EGYFIL™ is comparable to other drug-based topical treatments. Normalizing data to the relative t0, in patient with ankle pain, where non-surgical treatments are the first choice, in case of acute lateral sprains [54], long-term NRS reductions are higher than using a 1.3% diclofenac imbued patch: 22% vs 19% after 3 h, although lower after 3 days (42% vs 46%) [55]. Similarly, EGYFIL™ shows better pain relief than 1% topical diclofenac after 3 days of treatment (42% vs 26% normalized-NRS reduction) [56] but lower than 4% topical diclofenac [57]. Considering that the systemic effect of topical NSAIDs cannot be excluded [58] and considering the environmental pollution caused by these drugs [59], the use of EGYFIL™ HA-peptide mix represents a safe, reliable, and environmentally friendly alternative to soothe the pain.

Hyaluronic acid has been used successfully for the treatment of pain in a variety of pathologies, most notably in osteoarthritis and joint pain via intra-articular injection [19, 21, 60–63], however, based on our knowledge, no other clinical trial reports the usefulness of topical HA and peptides lotion to relieve muscle tension and pain. Puhl et al*.* showed that concentrations as low as 0.01% (0.25 mg/2.5 ml) of hyaluronate provided relief from OA pain [64], and results from other studies indicate that the analgesic effects of HA are not necessarily coupled to the lubricating or shock-absorbing actions of the macromolecule [21]. Indeed, Gomis et al. proposed that the elastoviscous properties of HA solutions are able to block the transmission of forces within the joint from passing to the stretch-activated channels in the nociceptor nerve terminals [21]. Something similar is potentially responsible for the observed effects in this study with EGYFIL™, where the peptides are dispersed into the hydrogel: SH-Polypeptide-6 and SH-Oligopeptide-1, were specifically selected to enhance the efficacy of HA in reducing discomfort and relieving pain. The skin, in particular the Stratum Corneum, represents a barrier for topical therapeutics, however, there are numerous strategies to overcome it; a variety of skin penetrating peptides have been documented which have the capacity to significantly increase the transdermal penetration of bound macromolecules [26, 65, 66]. The combination of these two peptides with HA seems to increase dermal penetration of this simple lotion without the need for excessive rubbing or massaging into the affected area, as well as to effectively cover and relieve a large surface area.

Additionally, topical application of HA and other large molecules can benefit through the use of penetration enhancers, such as glycerol, which has been shown to increase the transdermal penetration of HA almost 200 time more than HA in water [67].

This study has some limitations, in that it is a single arm study and thus cannot exclude the natural and physiological resolution of the symptoms over time. However, the data collected during the study highly suggests that the application of the lotion has a profound effect on reducing stiffness and pain in an expedited time frame, demonstrating that the lotion has a beneficial effect at quickly reducing discomfort over large body zones. Although acceptable from a statistics point of view, a further study into the mechanism of action, within a larger cohort of patients, including a control group, is warranted. Another limitation of the study is the absence of a follow-up later than three days, to verify if the positive effect of EGYFIL™ treatment continued once the application is interrupted. Finally, a more precise diagnosis before the treatment, could better identify the most suitable conditions for the alleviating efficacy of the lotion.

## Conclusions

The study herein shows that EGYFIL™ lotion appears to be safe and well tolerated by all patients exposed to its treatment. The reduction of both pain and stiffness over the course of the study, evaluated by Numerical Rate Score, revealed a quick response rate in relieving discomfort within three hours and significantly reduced pain and stiffness within three days, suggesting that the product can be effective for use in the treatment of stiffness and pain-related complaints in both sexes, in all age groups. No adverse reactions were recorded or observed throughout the study, denoting the product’s safety in repeated application.

### Supplementary Information

**Additional file 1. **

**Additional file 2: Supplementary Table 1. **Patients’ NRS pain and stiffness scores for each time-point.

## Data Availability

The datasets used and analysed during the current study is available from the corresponding author on reasonable request.

## Abbreviations

HA: Hyaluronic acid
SH: Synthetic Human
NF-kB: Nuclear Factor kappa B
TNF: Tumor Necrosis Factor
EGF: Epidermal Growth Factor
ICF: Informed Consent Form
IFU: Instruction For Use
NRS: Numerical Rating Scale
AE: Adverse Events
OA: Osteoarthritis
