# Supplementary material for: A pre-market interventional, single-arm clinical investigation of a new topical lotion based on hyaluronic acid and peptides, EGYFILTM, for the treatment of pain and stiffness in soft tissues
Source: BMC Musculoskelet Disord. 2023 Oct 2;24:777. doi: 10.1186/s12891-023-06903-y (PMC10544473; doi:10.1186/s12891-023-06903-y)
Supplement: Supplementary file 1 — Additional file 1. [file 12891_2023_6903_MOESM1_ESM.docx]

PAIN LOTION Questionnaire - **BASELINE**

| Initials: |  | Sex |  |
| --- | --- | --- | --- |
| Age: |  | Adverse Effects: |  |

1. Indicate on the diagram below where the PAIN LOTION is to be applied.
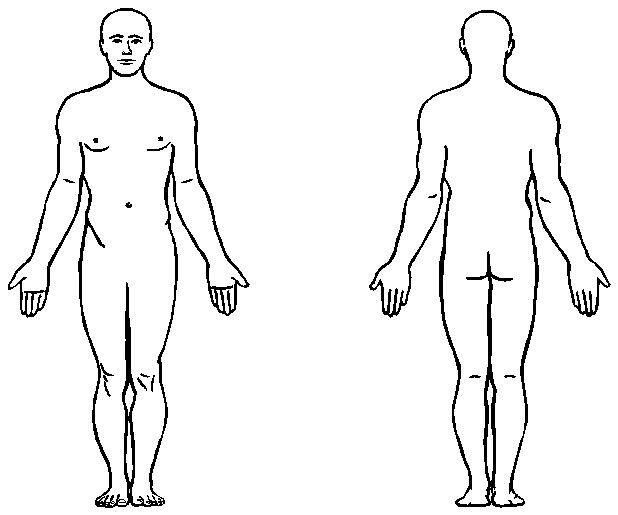

2. Are you applying the PAIN LOTION at the indicated points for **stiffness / pain / both**?
   if available, details of the type of pain can be noted here:______________________
3. IF PAIN: On a scale of 0-10 (where 0 is no pain and 10 is the worst pain), how bad is your pain currently, at rest, at the indicated point(s) above?
4. IF STIFFNESS: On a scale of 0-10 (where 0 is no stiffness and 10 is the worst stiffness), how bad was your stiffness most recently, at the indicated point(s) above?
5. Is the stiffness/pain indicated above due to sporting activity or not? **Y / N**
6. Are you taking or have you taken any pain medication in the last 12 hours? **Y / N**
   (This includes prescription drugs, OTC medication, herbal relaxants, etc.)
7. If Yes, please indicate which of the following.
   1. Herbal products
   2. OTC products
   3. Prescription medication
   4. Muscle relaxant
   5. None of the above/other

PAIN LOTION Questionnaire – **3 HOURS follow up**

| Initials: |  | Sex |  |
| --- | --- | --- | --- |
| Age: |  | Adverse Effects: |  |

1. IF PAIN: On a scale of 0-10 (where 0 is no pain and 10 is the worst pain), how bad is your pain currently, at rest, at the point(s) indicated at baseline?
2. IF STIFFNESS: On a scale of 0-10 (where 0 is no stiffness and 10 is the worst stiffness), how bad is your stiffness currently, at the indicated point(s) above?
3. How long after application of the PAIN LOTION did you start to feel relief
   (relief not disappearance of pain/stiffness)
   1. Within 5 minutes
   2. Within 10 minutes
   3. Within 15 minutes
   4. Within 30 minutes
   5. Greater than 30 minutes
   6. No relief
4. How long after the initial application did you feel the need to reapply the PAIN LOTION?
5. Within 30 minutes
6. Within 1 hour
7. Within 2 hours
8. Within 3 hours
9. No need to reapply
10. How many times did you re apply the PAIN LOTION in the past 3 hours?
11. Did this re-application sustain your relief (of pain and or stiffness) and improve your mobility? **Y / N** **/ NA**
12. Did you use other pain treatments since applying the PAIN LOTION? **Y / N**
13. If YES, indicate which of the following:
    1. Herbal products
    2. OTC products
    3. Prescription medication
    4. Muscle relaxant
    5. None of the above/other

PAIN LOTION Questionnaire – **3 DAYS follow up**

| Initials: |  | Sex |  |
| --- | --- | --- | --- |
| Age: |  | Adverse Effects: |  |

1. IF PAIN: On a scale of 0-10 (where 0 is no pain and 10 is the worst pain), how bad is your pain currently, at rest, at the point(s) indicated at baseline?
2. IF STIFFNESS: On a scale of 0-10 (where 0 is no stiffness and 10 is the worst stiffness), how bad is your stiffness currently, at the indicated point(s) above?
3. Did you use other pain treatments other than the PAIN LOTION in the past 3 days? **Y / N**
4. If YES, indicate which of the following:
   1. Herbal products
   2. OTC products
   3. Prescription medication
   4. Muscle relaxant
   5. None of the above/other
5. How long after the initial application did you feel the need to reapply the PAIN LOTION??
6. Within 6 hours
7. Within 12 hours
8. Within 24 hours
9. Within 2 days
10. Within 3 days
11. No need to reapply
12. How many times did you re apply the PAIN LOTION in the past 3 days?
13. Did this re-application sustain your relief (of pain and or stiffness) and improve your mobility? **Y / N** **/ NA**
14. Would you use this product for basic musculoskeletal complaints related to stiffness and moderate muscular pain? **Y / N**
15. How satisfied are you with the results you obtained from the PAIN LOTION?
    (not satisfied / somewhat satisfied / no opinion / satisfied / very satisfied)
16. How would you rate the feel of this product on your skin?
    (very poor / poor / no opinion / good / very good)
17. Would you recommend this product to others? **Y / N**
18. On a scale of 1-10 how would you rate the PAIN LOTION for the relief of passive stiffness?
    passive stiffness= stiffness from inactivity such as upon waking, long periods of sitting and /or no movement.
19. Would you use the PAIN LOTION on a regular basis for passive stiffness? **Y / N**
