# Supplementary material for: A pre-market interventional, single-arm clinical investigation of a new topical lotion based on hyaluronic acid and peptides, EGYFILTM, for the treatment of pain and stiffness in soft tissues
Source: BMC Musculoskelet Disord. 2023 Oct 2;24:777. doi: 10.1186/s12891-023-06903-y (PMC10544473; doi:10.1186/s12891-023-06903-y)
Supplement: Supplementary file 2 — Additional file 2: Supplementary Table 1. Patients’ NRS pain and stiffness scores for each time-point. [file 12891_2023_6903_MOESM2_ESM.docx]

| **Patient ID** | **Pain NRS t0** | **Pain NRS 3h** | **Pain NRS 3d** | **Stiffness NRS t0** | **Stiffness NRS 3h** | **Stiffness NRS 3d** |
| --- | --- | --- | --- | --- | --- | --- |
| 1 | 8 | 8 | 6 | 8 | 8 | 4 |
| 2 | 7 | 5 | 0 |  |  |  |
| 3 | 6 | 4 | 2 |  |  |  |
| 4 | 4 | 4 | 0 |  |  |  |
| 5 | 8 | 5 | 4 |  |  |  |
| 6 | 6 | 5 | 5 | 9 | 9 | 8 |
| 7 | 4 | 3 | 3 |  |  |  |
| 8 | 5 | 3 | 3 | 8 | 5 | 6 |
| 10 | 4 | 2 | 1 | 6 | 4 | 2 |
| 11 | 6 | 4 | 7 | 5 | 3 | 4 |
| 12 | 6 | 3 | 2 | 7 | 4 | 2 |
| 13 | 5 | 4 | 3 | 7 | 6 | 4 |
| 14 | 8 | 7 | 6 | 8 | 5 | 5 |
| 15 | 5 | 6 | 2 |  |  |  |
| 16 |  |  |  | 8 | 8 | 7 |
| 17 | 8 | 6 | 3 | 8 | 6 | 4 |
| 18 | 5 | 3 | 5 | 5 | 0 | 5 |
| 19 | 7 | 6 | 5 | 6 | 5 | 5 |
| 21 |  |  |  | 4 | 3 | 2 |
| 22 | 4 | 3 | 2 | 4 | 3 | 2 |
| 23 | 5 | 3 | 7 |  |  |  |
| 25 | 4 | 4 | 3 | 4 | 4 | 2 |
| 26 | 6 | 6 | 1 | 5 | 5 | 1 |

**Supplementary Table 1.** Patients’ NRS pain and stiffness scores for each time-point.
